# Supplementary material for: Silver Nanoparticle-Mediated Synthesis of Fluorescent Thiolated Gold Nanoclusters
Source: Nanomaterials (Basel). 2021 Oct 25;11(11):2835. doi: 10.3390/nano11112835 (PMC8624127; doi:10.3390/nano11112835)
Supplement: Supplementary file 1 [file nanomaterials-11-02835-s001.zip › nanomaterials-1409491-supplementary.pdf]

nanomaterials

Supplementary material

Silver Nanoparticle-Mediated Synthesis of Fluorescent Thiolated Gold Nanoclusters

Cheng-Yeh Chang †, Tzu-Hsien Tseng †, Bo-Ru Chen, Yi-Ru Wu, Cheng-Liang Huang and Jui-Chang Chen *

Department of Applied Chemistry, National Chiayi University, Chiayi City 600355, Taiwan; sss1072727@gmail.com (C.-Y.C.); tzuhome50@gmail.com.tw (T.-H.T.); s1090251@mail.ncyu.edu.tw (B.-R.C.); clhuang@mail.ncyu.edu.tw (C.-L.H.); chenjc@mail.ncyu.edu.tw (J.-C.C.)
* Correspondence: chenjc@mail.ncyu.edu.tw; Tel.: +886-5-2717968 Fax: +886-5-2717901
† These authors contributed equally to this work.

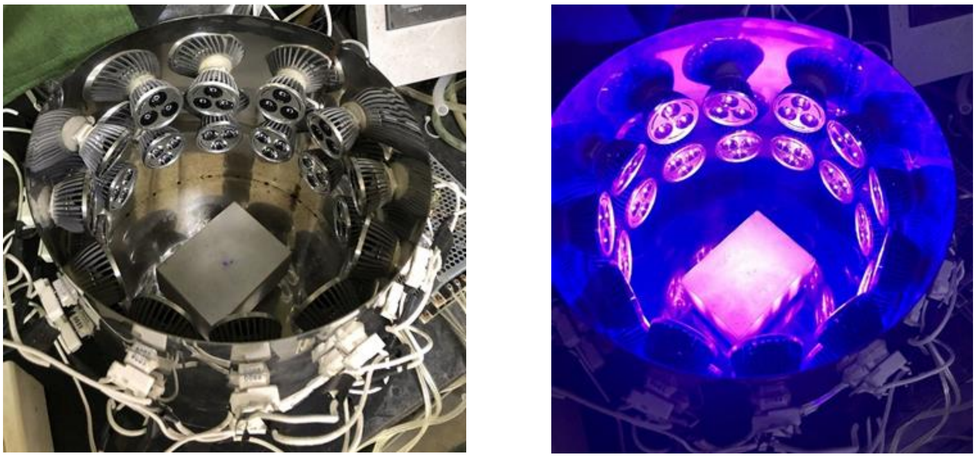

Figure S1. The setups for violet LED irradiation to generate quasi-spherical Ag NPs. The cabinet, containing 24 LEDs with 405 nm wavelengths, was used for uniform light intensity (0.01 W/cm^2), surrounding a 50-mL reaction vial. The LED lamps were turned off (**left**), and on (**right**).

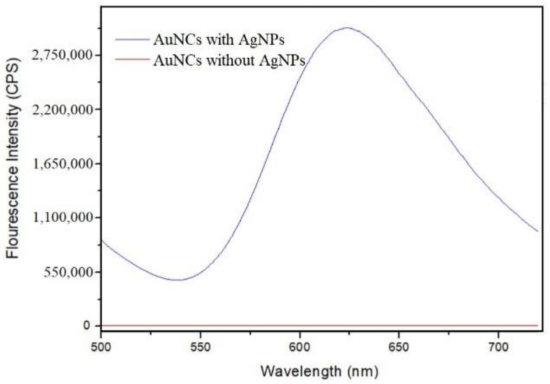

Figure S2. Emission scan of samples from solutions for synthesis of Au NCs in the presence (blue line), and absence (red line), of Ag NPs. The excitation wavelength was set at 370 nm.

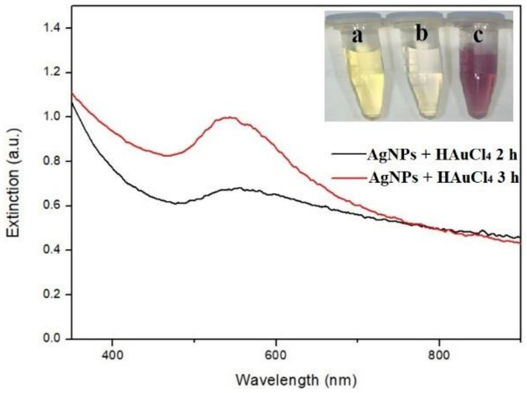

Figure S3. Time-dependent UV-Visible spectroscopy of solution after Ag NPs were mixed with $HAuCl_4$. Upper panel: (**a**) Ag NPs solution only; (**b**) right after the Ag NPs were mixed with $HAuCl_4$; and **(c)** 3 h after the Ag NPs were mixed with $HAuCl_4$.

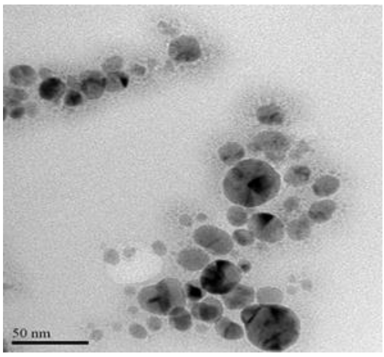

Figure S4. TEM images of quasi-spherical Ag NPs after they were mixed with GSSG.
